# Editorial: Current advances in paediatric bronchiectasis: from early childhood prevention to transition to adult care

**DOI:** 10.3389/fped.2023.1336029

**Published:** 2023-12-06

**Authors:** G. B. McCallum, J. M. Marchant, V. Goyal

**Affiliations:** ^1^ Child and Maternal Health Division, Menzies School of Health Research, Charles Darwin University, Darwin, NT, Australia; ^2^ Department of Respiratory and Sleep Medicine, Queensland Children’s Hospital Queensland University of Technology, Brisbane, QLD, Australia

**Keywords:** bronchiectasis, transition, chronic cough, pediatric care, management, exercise, trial, physiotherapy

**Editorial on the Research Topic** Current advances in paediatric bronchiectasis: from early childhood prevention to transition to adult care

While once considered rare, the importance of bronchiectasis has been increasingly appreciated (1), and is now recognised in all settings globally (2–4). Currently, the prevalence of childhood bronchiectasis remains disproportionally higher among socially disadvantaged populations of high-income countries (e.g., Australian First Nations children from the Northern Territory [NT] [1 in 68] (5), Alaskan Natives [1 in 63]) (6), and in low-middle income countries (3), although it is increasing in non-First Nations children and adults in high-income countries (2, 3, 7) Annual hospitalization rates in children < 15 years of age had a greater than fourfold increase between 2000 and 2017 in New Zealand (8). Importantly, in adults with bronchiectasis, > 60% had symptoms present from childhood (9). Although our understanding of bronchiectasis is improving, we still have a long way to go to address important clinical and management gaps extending from paediatric to adult care. However, there remains a lack of funding, resource allocation and coordinated, interdisciplinary care globally for childhood bronchiectasis. Identifying novel, evidence-based solutions to improve the management of bronchiectasis from childhood through to adulthood will undoubtably improve both short- and long-term clinical outcomes. This special issue provides a collection of six new research articles drawing on work of researchers globally from three countries. Each article broadens our knowledge on possible underlying causes and the management among children/adolescents with bronchiectasis.

Bronchiectasis is a heterogenous disease with multiple aetiologies and risk factors, that vary by region (2). Determining the aetiology or contributing factors to developing bronchiectasis (10) is however important, as this may identify treatable traits to directly inform management. Zhu and Jin, report a case study of a seven year old boy, whose bronchiectasis was identified inadvertently by a scan of the abdomen on computed tomography. Through further medical history, investigations, and genetic testing for hearing loss due to Pendred syndrome, a heterozygous mutation in the *SLC26A4* gene was identified which can lead to reduced airway defence, chronic inflammation, destruction of airway wall structure and bronchiectasis (11). Although speculative, the authors hypothesised a possible association between the *SLC26A4* gene and bronchiectasis in this child, urging clinicians to consider possible links.

Verwey et al. provide a commentary into the paucity of paediatric data on bronchiectasis in Africa and the challenges, barriers, and risk factors to providing early and optimal care. They highlighted the need for further research and resources to address known clinical gaps and the unmet needs of children in Africa with bronchiectasis to determine underlying aetiology, improved diagnosis, and management pathways to inform clinical practice and policy. They also describe the importance of extending their bronchiectasis registry to a global platform to facilitate further research and improved understanding of bronchiectasis among families through education that meets the local needs of the community.

The aim of bronchiectasis management in children/adolescents is to “(1) optimise lung growth, (2) preserve lung function, (3) optimise quality of life, (4) minimise exacerbations, (5) prevent complications and 6) if possible, reverse structural lung injury” (1). The remaining articles in this edition describe different aspects of managing childhood bronchiectasis.

Improving disease monitoring has been recently identified as a major clinical and research priority for children and adolescents with bronchiectasis (12). In their article, Ramsey and Schultz describe methods to monitor disease progression that include a range of modalities including lung imaging, respiratory function, patient-reported outcomes, respiratory exacerbations, sputum biomarkers and nutritional outcomes. They recognise potential challenges, enablers, and barriers in achieving these outcomes in settings where access to specialist services and resources are limited and highlight the need for greater investment into development of non-invasive measures to monitor disease progression in childhood bronchiectasis.

Despite guidelines recommending regular exercise to improve cardiovascular fitness and quality of life (1), children with bronchiectasis have inadequate levels of physical activity (13). In this study from Australia, Joschtel et al. describe a pilot randomised controlled trial among 21 children with bronchiectasis that assessed the efficacy of a play-based exercise program over seven weeks to improve fundamental movement skills and fitness among children with bronchiectasis. This trial found that cardiovascular fitness improved over the trial period, but no improvement was found for perceived competence and/or quality of life. They concluded larger trials over a longer duration are required to determine whether results are maintained over time.

Furthermore, regular physiotherapy is recommended as part of multidisciplinary management of bronchiectasis (1, 14). Welford et al. undertook a retrospective review among 143 First Nations children with bronchiectasis from remote communities of the Top End of the NT to examine physiotherapy management at diagnosis and in the following 12 months in the community. They found that at diagnosis, only two-thirds of children received physiotherapy interventions in line with current guidelines, however in the following 12 months, physiotherapy management was very poor with only 5.5% having evidence of referral and only 7.7% being clinically reviewed.

With increasing survival of young people with chronic diseases into adulthood, awareness of the transition process to improve clinical outcomes from paediatric to adult services has been recognised (15). The final article by Schutz et al. also from the NT, describe what processes, timeframes and supports were in place for the transition of young people with bronchiectasis to adult care. Among 102 young people (aged ≥ 14 years), only 8.8% had some form of documented evidence of transition planning but no there was no evidence of any young person attending adult respiratory clinics.

Both articles from the NT (Welford et al., Schutz et al.) demonstrate a significant gap in the delivery of care for children/young people with bronchiectasis living in remote communities, settings with the highest rates globally. Therefore, there is urgent need to develop evidence-based standardized pathways to optimise management in these settings. Importantly, suboptimal management may be associated with poorer clinical outcomes and premature death as is seen in First Nations adults with bronchiectasis from Central Australia of the Northern Territory, where the gap in life expectancy is >20 years compared to other non-First Nations adults with bronchiectasis (16).

While progress has been made on paediatric bronchiectasis in recent times, further investment and funding, resource allocation and coordinated, interdisciplinary care is needed globally to address known clinical and management gaps from paediatric extending to adult care.
